# Are a lack of social relationships and cigarette smoking really equally powerful predictors of mortality? Analyses of data from two cohort studies

**DOI:** 10.1016/j.puhip.2021.100140

**Published:** 2021-05-11

**Authors:** G. David Batty, Paola Zaninotto, Marko J. Elovainio, Christian A. Hakulinen

**Affiliations:** a Department of Epidemiology and Public Health, University College, London, UK; b School of Biological and Population Health Sciences, Oregon State University, Corvallis, USA; c Department of Psychology and Logopedics, University of Helsinki, Helsinki, Finland

**Keywords:** Social isolation, Loneliness, Smoking, Mortality

## Abstract

**Objective:**

The suggestion from cross-review comparison that lower levels of social integration (social isolation, loneliness) and cigarette smoking are equally powerful predictors of premature mortality has been promulgated by policy organisations and widely reported in the media. For the first time, we examined this assertion by simultaneously comparing these associations using data from two large cohort studies.

**Study design:**

Individual-participant analyses of two large prospective cohort studies.

**Methods:**

Participants in UK Biobank and the English Longitudinal Study of Ageing reported loneliness, social-isolation and smoking behaviours using standard scales at baseline. Cause-specific mortality was ascertained via linkage to national registries. We used Cox regression analyses to compute a relative index of inequality to summarise the relation between these baseline characteristics and mortality experience.

**Results:**

Mean age at baseline was 56.5 years in the 466,876 (273,452 women) Biobank participants and 66.1 years in the 7505 (4123 women) English Longitudinal Study of Ageing members. In Biobank, a mean duration of mortality surveillance of 6.6 years gave rise to a total of 13,072 deaths, while in the English Longitudinal Study of Ageing, 1183 deaths occurred after a mean of 7.7 years. In ascending magnitude, loneliness, social isolation then cigarette smoking were associated with an increased risk of mortality from all-causes and all cancers combined. When cardiovascular disease mortality was the endpoint of interest, both smoking and social isolation, though not loneliness, revealed similar relationships.

**Conclusions:**

Contrary to cross-review comparisons, in the present datasets it appears that poor social integration is in fact less strongly linked to total mortality than cigarette smoking.

## Highlights

• Low levels of social integration have been compared to cigarette smoking as an equally powerful risk factor for mortality.
• We empirically examined this assertion by simultaneously comparing these associations using data from two cohort studies.
• Poor social integration was in fact less strongly linked to mortality than cigarette smoking.

It is well established that individuals who have lower levels of social integration experience an increased risk of premature mortality [1,2]. The reviews on which these associations are based, however, also posit that the magnitude of this association is the same as that for risk factors known to be causally linked to mortality, such as cigarette smoking [1,2]. This assertion – enthusiastically promoted by policy organisations and widely reported in the media – is, however, based on cross-review comparisons rather than direct evaluation within the same study. Accordingly, for the first time to our knowledge, we compared the magnitude of individual associations of social support, loneliness, and cigarette smoking with total and cause-specific mortality using individual-participant data from two prospective cohort studies.

We used data from UK Biobank [3,4] (baseline 2006–10) and the English Longitudinal Study of Ageing (ELSA) (baseline 2002–03) [5,6], on-going prospective cohort studies with detailed and comparable information on social isolation, loneliness, and cause-specific mortality. In UK Biobank, ethical approval was received from the North-West Multi-centre Research Ethics Committee, and the research was carried out in accordance with the Declaration of Helsinki of the World Medical Association. In the English Longitudinal Study of Ageing, data collection was approved by the London Multicentre Research Ethics Committee. No specific ethical approval was required for the present analyses of anonymised data.

In ELSA, loneliness was measured using the UCLA loneliness scale [7], while in Biobank selected questions from this scale were utilised. Ratings were summed (ELSA range: 0–9; Biobank range: 0–2), with a higher score indicating greater loneliness. Social isolation in ELSA was based on responses to enquiries regarding marital/cohabiting status; contact with children, other family members, or friends; and participation in organisations such as clubs or religious groups. Similar questions were asked of study members in Biobank, including the number of people sharing the study members household; frequency of visits with friends or family; and engagement in leisure/social activities. Ratings were summed (ELSA range: 0–5; Biobank range: 0–3), with a higher score denoting greater isolation.

In both studies, smoking behaviour was based on standard enquiries and mortality, including cause of death, was ascertained by linkage of study members with a national registry (ending 16th February 2016 for Biobank; 14^th^ February 2013 for ELSA). Given that the isolation, loneliness and smoking variables have different scales, for a direct comparison of the magnitude of the relation of each with mortality, we used the relative index of inequality (RII) [[8], [9], [10]]. The RII is derived by ranking the subjects on each of the exposure variables and then dividing this score by the sample size to yield a value between 0 and 1. When included in Cox proportional hazard models, the RII can be interpreted as the mortality hazard between the extreme ends of the risk factor distribution. Thus, an RII of 2.0 indicates that the mortality hazard is twice as high for the most disadvantaged relative to the most advantaged.

Mean age at baseline was 56.5 years in the 466,876 Biobank participants (273,452 women) and 66.1 years in the 7505 ELSA members (4123 women). Around ninety-five percent of each sample was ethnically white. In Biobank, a mean duration of mortality surveillance of 6.6 years gave rise to a total of 13,072 deaths, while in the English Longitudinal Study of Ageing, 1183 deaths occurred after a mean of 7.7 years.

**Fig. 1:**
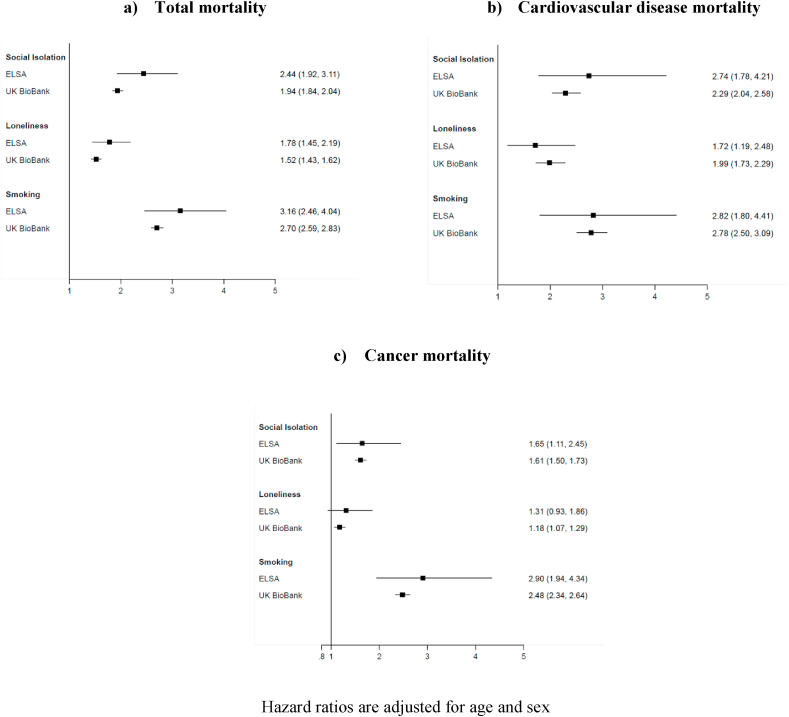
Relative index of inequality (95% confidence interval) for the association of social isolation, loneliness, and cigarette smoking with mortality from all-causes, cardiovascular disease, and cancer.

In Fig. 1, we show the age- and sex-adjusted RII for social isolation, loneliness, and cigarette smoking in relation to three principal mortality outcomes. In ascending magnitude, loneliness, social isolation then cigarette smoking were all associated with an increased risk of total mortality (Fig. 1a) in both studies. When cardiovascular disease mortality was the endpoint of interest (Fig. 1b), both smoking and social isolation, though not loneliness, revealed similar relationships. This was not the case, however, for death from cancer of all sites combined, where, in keeping with the analyses of total mortality, cigarette smoking was markedly more strongly associated with disease risk than either of the two social variables (Fig. 1c).

We found that, of the characteristics of social isolation, loneliness, and cigarette smoking, smoking was typically more hazardous to physical health when based on mortality outcomes. This discordance was most pronounced for cancer and total mortality but less so for cardiovascular disease. Thus, contrary to cross-review comparisons, it seems that poor social integration is in fact less strongly linked to mortality than cigarette smoking. Our findings warrant testing in other cohort studies.

## Funding

GDB is supported by the UK 10.13039/501100000265Medical Research Council (MR/P023444/1) and the US 10.13039/100000049National Institute on Aging (1R56AG052519-01; 1R01AG052519-01A1).

## Access to data

Access to data from UK Biobank (http://www.ukbiobank.ac.uk/) and English Longitudinal Study of Ageing (https://data-archive.ac.uk/) is available upon application.

## Declaration of competing interest

The authors declare that they have no known competing financial interests or personal relationships that could have appeared to influence the work reported in this paper.
